# Diversity in Genetic Regulation of Bacterial Fimbriae Assembled by the Chaperone Usher Pathway

**DOI:** 10.3390/ijms24010161

**Published:** 2022-12-22

**Authors:** Dharmender K. Gahlot, Nayyer Taheri, Sheila MacIntyre

**Affiliations:** 1 School of Biological Sciences, University of Reading, Reading RG6 6EX, UK; 2 Department of Molecular Biology, Umeå University, 901 87 Umeå, Sweden; 3 Umeå Centre for Microbial Research (UCMR), Umeå University, 901 87 Umeå, Sweden; 4 APC Microbiome Institute, University College Cork, T12 K8AF Cork, Ireland

**Keywords:** Gram-negative bacteria, Chaperone Usher Pathway (CUP) fimbriae, FGL & FGS class, expression regulation mechanisms, lifestyle & pathogenesis, vaccine development

## Abstract

Bacteria express different types of hair-like proteinaceous appendages on their cell surface known as pili or fimbriae. These filamentous structures are primarily involved in the adherence of bacteria to both abiotic and biotic surfaces for biofilm formation and/or virulence of non-pathogenic and pathogenic bacteria. In pathogenic bacteria, especially Gram-negative bacteria, fimbriae play a key role in bacteria–host interactions which are critical for bacterial invasion and infection. Fimbriae assembled by the Chaperone Usher pathway (CUP) are widespread within the *Enterobacteriaceae*, and their expression is tightly regulated by specific environmental stimuli. Genes essential for expression of CUP fimbriae are organised in small blocks/clusters, which are often located in proximity to other virulence genes on a pathogenicity island. Since these surface appendages play a crucial role in bacterial virulence, they have potential to be harnessed in vaccine development. This review covers the regulation of expression of CUP-assembled fimbriae in Gram-negative bacteria and uses selected examples to demonstrate both dedicated and global regulatory mechanisms.

## 1. Introduction

Fimbriae or pili are hair-like proteinaceous appendages that are present on the surface of many commensal and pathogenic bacteria [1,2,3]. They are primarily involved in the adherence of bacterial cells to both abiotic and biotic surfaces and thus have an important role in biofilm formation, initiation of infection via specific host cell binding and in some cases invasion [2,4,5,6,7]. Pili have been classified according to the mechanism by which they are assembled. In Gram-negative bacteria, in addition to conjugative pili, this includes Chaperone-Usher Pathway (CUP) assembled pili [3,8,9,10], Type IV (-4) pili [1,3,11,12], Curli fibres [13,14], pili involved in Type IV secretion system (T4SS) [15] and, in Gram-positive bacteria, sortase assembled pili [1,3,6]. A consequence of the assembly mechanism and final structure of these organelles, is that some pili have other functions in addition to adhesion. Type IV pili are associated with twitching motility via the ability to retract pili and Type IV pili, conjugative pili and T4SS are all involved in DNA transfer. In addition, surface pili, also called needles, of T4SS and Type 3 secretion system (T3SS) transfer effector proteins [1,15,16,17]. In the *Enterobacteriaceae*, pili/fimbriae assembled by the CU pathway are prevalent [8]. While the term pili often refers to more structured, longer hair-like appendages, and fimbriae to thinner structures, within the CU pathway both terms have been used. The ubiquitous nature of these fimbriae, and differences in binding specificities involved, highlight the crucial role that these structures have in the pathogenesis of many bacterial species [5,8].

Expression of fimbrial operons is generally tightly regulated and fimbriae are often only expressed under specific environmental conditions [8,18,19,20]. This is essential to ensure effective niche colonisation while optimising immune evasion. This review focuses on the expression of fimbriae assembled by the CU pathway, highlighting the diversity of regulatory mechanisms. The review mainly considers the primary level of regulation, which frequently involves regulatory genes directly linked to the fimbrial cluster but in some cases involves control by unlinked gene products, global virulence regulators. Based on sequence analysis and predicted structures, these regulators belong to different families corresponding to the well-studied regulators, AraC/XylS (A/X), LysR, CRP, and OmpR [21]. In addition to responding to environmental factors, regulatory cross-talk has been identified between different fimbrial clusters and also between different physiological process, such as adhesion and swimming. Using selected examples, primarily from the *Enterobacteriaceae,* common features of CUP fimbriae and their assembly are briefly introduced (for detailed reviews see [8,16,22,23,24] prior to consideration of the diverse mechanisms that control fimbriae expression.

### 1.1. Fimbriae: Genetic Clusters and Assembly via the CU Pathway

Assembly of CUP fimbriae has been extensively studied in strains of uropathogenic *Escherichia coli* (UPEC), one of the most common causative agents of the urinary tract infection (UTI) in humans [8,16,17,18,19,22]. Type 1 fimbriae bind mannose residues and in UTI bind to uroplakins in the bladder epithelium. Pap pili bind to the diasaccharide Galα(1-4)Gal linkage on P blood group cells and are important in kidney colonisation and infection. The detailed characterisation of assembly of Pap pili and type 1 fimbriae from these bacteria has highlighted the requirement of a dedicated periplasmic chaperone and outer membrane usher protein for synthesis of each different fimbriae [16,20,22,23,24,25,26,27,28,29,30,31,32]. Surface assembly of CUP fimbriae requires specific binding of the nascent fimbrial subunits by the cognate chaperone in the bacterial periplasm via a process known as donor-strand complementation [23,25,26,27,28]. In this step, a chaperone: subunit complex is formed in which the chaperone G1 β–strand complements a hydrophobic cleft in the partially folded subunit, thus stabilising an assembly competent intermediate. Subsequent targeting of the subunit: chaperone complex to the outer membrane usher is followed by polymerisation of the fimbrial subunits, involving periplasmic domains of the usher protein [23,29,30,31]. Polymerisation is achieved as the N-terminus of an incoming fimbrial subunit displaces the chaperone G1 β–strand to form a non-covalent, but highly stable subunit: subunit interaction, a process termed donor-strand exchange [23,28,29,30,31,33]. Assembly and transfer through the outer membrane usher to form surface located organelles is driven by these protein-protein interactions [30,31,32,33]. With complex pili, such as Pap and type 1 pili, assembly of adhesin and subunits occurs in a highly ordered fashion [8,23,31].

**Figure 1:**
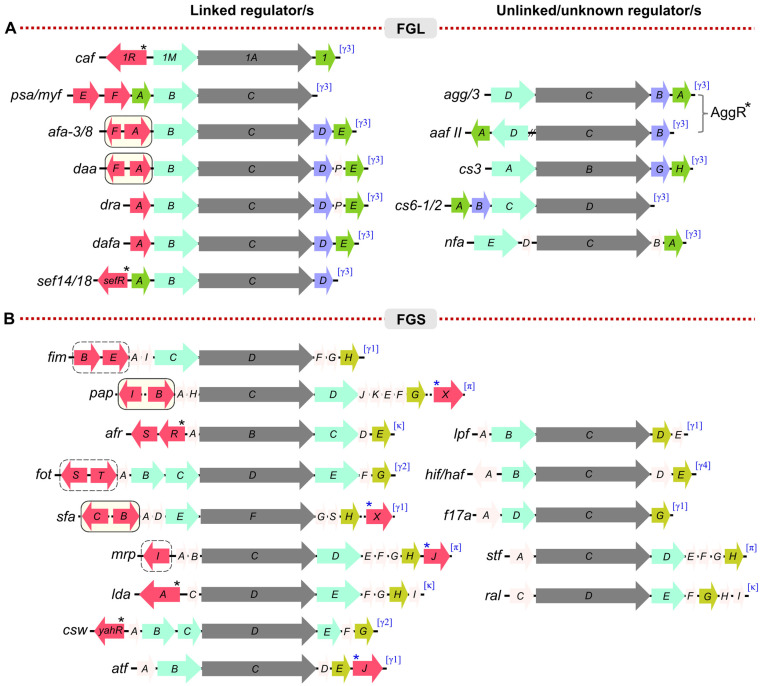
Diversity in gene-cluster of the CUP assembled fimbriae of Gram-negative bacteria. Selected fimbrial clusters and regulator genes of the structurally and functionally distinct groups, FGL (**A**) and FGS (**B**), where the F_1_-G_1_ loop of the chaperone is either Long (as in FGL) or Short (as in FGS). Coloured arrows indicate the orientation of each gene encoding; red (regulator/s where identified), cyan (periplasmic chaperone), grey (outer membrane usher), apple green (adhesin, FGS), jade green (major subunit/adhesin, FGL), purple (additional subunit/invasin, FGL), light pink, additional pilin subunits (FGS) or protein/s of unknown function (FGL). DNA binding regulators belonging to the A/X family of transcriptional factors are indicated by black (*) asterisk, with blue asterisk indicating *mrpJ* homologues. Regulation by recombinases/promoter inversion is depicted in enclosed dashed boxes. Epigenetic regulation, via Dam-methylation, is indicated by beige boxes with solid lines. Gene clusters are from *Escherichia coli*, except *caf, psa* (*Yersinia pestis*); *lpf*, *sef, stf* (*Salmonella* spp.); *mrp*, *atf* (*Proteus mirabilis*); *hif*/*haf* (*Haemophilus influenzae*). Gene clusters originally sourced from Zav’yalov et al. [24], and adapted [8,18,36,38,39,40,41,42,43,44,45].

Genes encoding the CUP fimbriae are organised in operons or gene clusters, which can be located either on the bacterial chromosome or on a plasmid [8,34,35]. It has been demonstrated that a single bacterial genome can have multiple fimbrial operons dispersed throughout the genome [35]. A survey of *E. coli* strains identified an average of 12 fimbrial operons per strain [35] and 17 fimbrial operons have been identified in *Proteus mirabilis* [36]. Functional operons always contain a gene for the major structural subunit, a periplasmic chaperone, and an outer membrane usher (Figure 1). Most contain additional genes encoding structural proteins, such as minor subunits and terminal adhesins, a few include additional chaperones, and genes encoding regulatory proteins have been identified linked to a number of CUP loci [8,18,24,37].

### 1.2. Diversity in the CUP-Assembled Fimbriae

The CUP fimbriae operons were broadly divided into two structurally and functionally distinct groups, FGL and FGS (Figure 1) [46,47]. In the FGL group, fibre assembly is assisted by a cognate chaperone with an extended donor G1 β–strand that correlates with a longer F_1_–G_1_ loop (FGL) and high affinity for the subunit. Well-studied examples of the CUP fimbriae from the FGL group include the F1 capsular antigen [26,27,28,29,30,38,48,49] and Psa fimbriae of *Y. pestis* [50,51] and the Afa/Dra family of fimbriae from *E. coli* [8]. Most of the FGL-grouped fimbriae assemble into simple, thin (~2–3 nm diameter), and flexible fibres composed of only one or two subunit types (Figure 1). In contrast, fimbriae (pili) belonging to the well-defined FGS group are generally thicker (~7 nm diameter) and more rigid. These fimbriae have a more complex structure with a major structural pilin subunit that constitutes the shaft of the pilus plus several additional subunits which form a thin, flexible tip fibrillum capped by a single specialised adhesin. This terminal adhesin typically has two domains, both a pilin and an adhesin domain and hence is approximately twice the size of pilin subunits [17,24]. Thus, chaperones of FGS (F_1_–G_1_ short loop) fimbrial clusters have a requirement to recognise a number of different subunits and hence a lower specificity of binding to each subunit. Hence, the subgrouping based on chaperone properties, appears to correlate with complexity of fimbrial structure. Phylogenetically, based on usher sequence, the FGS group is subdivided into several clades including, β-, γ1-, γ2-, γ4-, κ-, and π-fimbriae [37]. Unlike FGS, the FGL group is a small monophyletic group, having only one clade, γ3. The α- clade corresponds to CU systems defined as ‘alternate’ CUP, including colonisation factor CS-1 of enterotoxigenic *E. coli* (ETEC) [37]. The following sections of the review, discuss knowledge of the regulatory mechanisms of expression of some well-characterised examples of CUP fimbriae.

## 2. Diversity in Regulation: Phase Variation

### 2.1. Regulation of Type 1 Fimbriae Expression: Promoter Inversion

**Figure 2:**
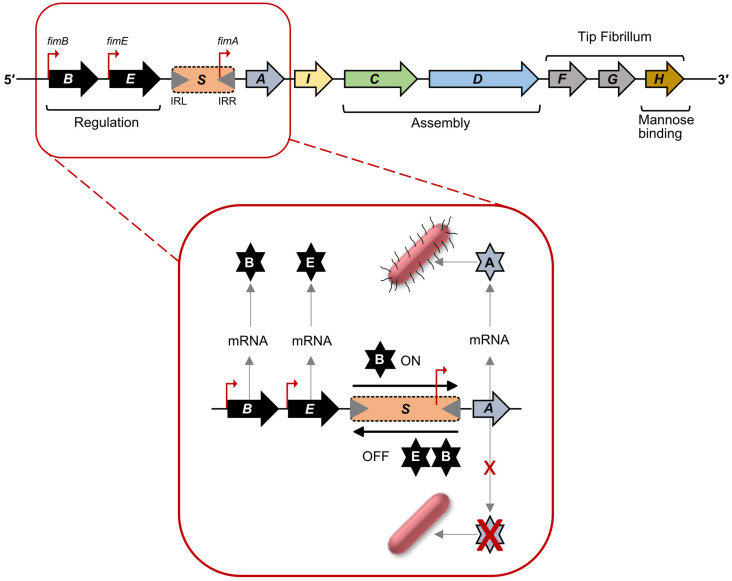
Regulation of type 1 fimbriae expression: promoter inversion. The *fim* operon indicating key features, promoters and regulatory invertible *fimS* DNA element. The *fimS* is flanked by inverted repeats IRL and IRR which are responsible for Phase ON/OFF states. Regulation of the *fim* operon is illustrated, as pull-out, with FimB- and FimE-mediated inversion of *fimS*. The FimB influences ON state and piliated bacteria. FimB and FimE both bind to influence switch to non-piliated bacteria.

Type 1 fimbriae of UPEC (FGS-grouped fimbriae) are synthesised from a chromosomally encoded gene cluster known as the *fim* locus. This locus is composed of nine genes, encoding two regulatory proteins, FimB and FimE; a major subunit pilin, FimA; two assembly proteins including a periplasmic chaperone, FimC and an outer membrane usher, FimD; two tip-associated fibrillum subunits, FimF and FimG; a mannose-binding adhesin, FimH, and finally FimI, a subunit responsible for anchoring the pilus to the cell surface (Figure 2) [8,9,25,46,52,53,54]. Expression of the *fim* locus is controlled by phase variation mediated by inversion of the promoter on a 314 bp DNA fragment, known as *fimS,* flanked by 9 bp inverted repeats, IRL and IRR (TTGGGGCCA) (Figure 2). When switched to the ON orientation, the *fimA* promoter leads to transcription and synthesis of the major fimbrial subunit, FimA, plus other structural subunits and assembly genes. In the opposite orientation the *fimA* promoter is non-functional. Hence, the forward orientation leads to Phase-ON (piliated state), and reverse orientation leads to Phase-OFF (non-piliated state). The regulatory proteins, FimB and FimE influence the orientation of *fimS*. These are site-specific recombinases, share 48% amino acid identity, and contain a tetrad of conserved amino acids (Arg47, His141, Arg144 and Tyr176; in FimB and Arg41, His136, Arg139 and Tyr171; in FimE), which has been shown to be essential for recombinase activity [52,53,54,55,56,57]. Both bind to regions flanking *fimS* including the inverted repeats.

FimB can influence the switch of *fimS* to either Phase-ON or Phase-OFF, while presence of FimE primarily leads to a switch of *fimS* to the Phase-OFF state (Figure 2). The mechanism and factors influencing the orientation and recombination of the *fimS* element is not only influenced by the concentrations of FimB and FimE, but also by other factors. Three promoters have been identified for *fimB* expression [58,59,60], while for *fimE* a single promoter has been identified [60].

**Figure 3:**
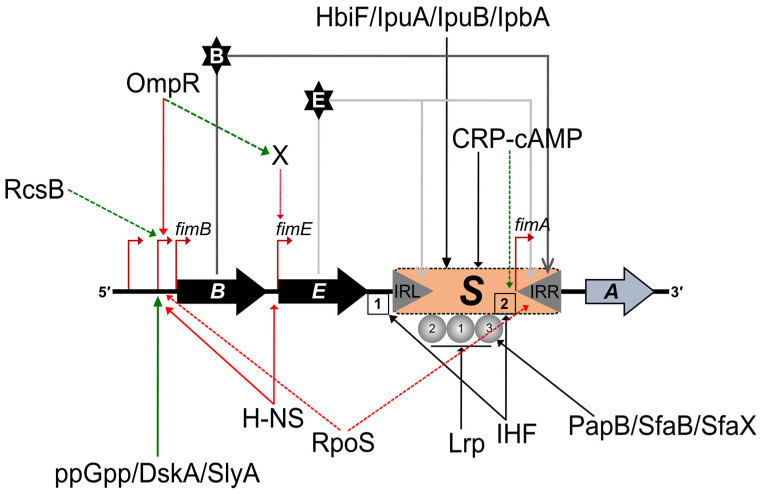
Auxiliary proteins known to influence the expression of type 1 fimbriae. A schematic of function/s of key auxiliary proteins in regulation of type 1 fimbriae is depicted. Invertible motifs, IRL and IRR within the *fimS* switch are indicated by grey triangles. IHFs (1, 2) and Lrp (1–3) binding sites are represented as open boxes and grey-coloured balls, respectively. Orientation and location of the *fimB*, *fimE* and *fimA* genes are also displayed. The identified promoters are shown as bent red arrows. Confirmed and presumed binding (of auxiliary proteins) associated with stimulatory effects is presented by solid and dashed green arrows, respectively; whereas confirmed and presumed binding (of auxiliary proteins) associated with repressing effects are shown by solid and dashed red arrows, respectively. Diagram is based on information from Schwan [54].

At least 20 additional auxiliary genes are known to influence the expression of type 1 fimbriae (Figure 3) [54]. The product of *pilG,* an allele of the *hns* gene, was reported to influence inversion; a mutation in the *pilG* was shown to increase inversion of *fimS* up to 100-fold using a *fimA*-*lacZ* fusion [61]. The global regulatory protein, H-NS (histone-like nucleoid-structuring protein) was shown to repress transcription of both *fimB* and *fimE* genes by binding to promoters of both with high specificity [54,58,60]. H-NS may also directly affect inversion by binding to sequences adjacent to the *fimS* DNA fragment. In addition to H-NS, integration host factor (IHF) and leucine-responsive protein (Lrp) also influence phase variation [62]. Other site-specific recombinases (HbiF, IpuA, IpuB and IpbA) have been identified as possibly influencing inversion of *fimS*. There is also crosstalk between regulators of different pili in *E. coli*. PapB, a regulatory protein of the Pap pili locus, and SfaB from the S pili locus can influence the orientation of *fimS* by inhibiting the Phase-OFF to the Phase-ON switch, enhancing selective expression of the correct pili at the right time and place. The alarmone, ppGpp also appears to be required for optimum expression of type 1 fimbriae, and the ppGpp alarmone, along with RNA polymerase-binding protein, DksA was shown to stimulate transcription of *fimB* from the P2 promoter [54]. Moreover, environmental factors such as temperature and pH also play a crucial role in the expression of type 1 fimbriae. Several studies have shown that the phase switching from ON to OFF state surges at a lower temperature [54,63,64]. Under low pH conditions type 1 fimbriae production is inhibited and increases at neutral pH. At neutral pH, proteins such as SlyA or RcsB may promote type 1 fimbriae synthesis by activating *fimB* and preventing H-NS binding [54].

Phase variation via promoter inversion has been shown to control expression of several other fimbriae, including CS18 fimbriae from enterotoxigenic *E. coli* (ETEC) and mannose-resistant pili (MR/P) from *Proteus mirabilis* (Figure 1). The *fot* cluster, encoding CS18 fimbriae, is regulated by a 312 bp inversion driven by the two site-specific recombinases, FotS and FotT, that share close to 50% sequence identity with FimB and FimE, respectively [39]. Similar sequence identity is not shared between other genes of the two operons. The *mrp* cluster is controlled by promoter inversion on a 252 bp sequence, flanked by inverted repeats and located between *mrpI* and *mrpA*, which encodes the major subunit of MR/P fimbriae. MrpI also shares high homology with FimB and E, but unlike the *fim* and *fot* clusters, MrpI is the sole recombinase [40,41]. It is specific for the *mrp* promoter switch, and can also switch the promoter to either ON or OFF position. Expression of MR/P fimbriae is strongly upregulated in vivo. MrpJ, a MarR-like transcription factor and product of the last gene of the *mrpABCDEFGHJ* operon (Figure 1) can function as an auto-activator of the operon [42]. Detail of how promoter inversion, *mrpJ* expression and environmental factors interact to drive promoter orientation to the ON- phase remain to be elucidated.

*P. mirabilis*, a significant pathogen of urinary tract infections, is highly motile and exhibits characteristic swarming activity. During infection co-ordination between bacterial swimming and adhesion is critical. In accord with cross-talk between expression of fimbrial operons, MrpJ has been shown to decrease flagella production and swimming by repressing transcription of the flagellar master regulator, *flhD* [36,42]. Recent studies have outlined an MrpJ regulon, involving cross-talk between fimbrial operons, flagella expression and the Type-6 secretion system [42]. The *mrpJ* gene is present in 10 of the 17 fimbrial operons of *P. mirabilis*, including the *atf* locus, shown in Figure 1 [36], and homologues have been identified adjacent to the *E. coli sfa* locus, encoding S pili associated with infant meningitis, and also downstream from the *pap* operon in some *E. coli* strains (Figure 1). PapX similarly decreases flagella production by binding to the *flhD* promoter [43,65].

### 2.2. Regulation of Pap pili Expression: Methylation/Epigenetic Switch

**Figure 4:**
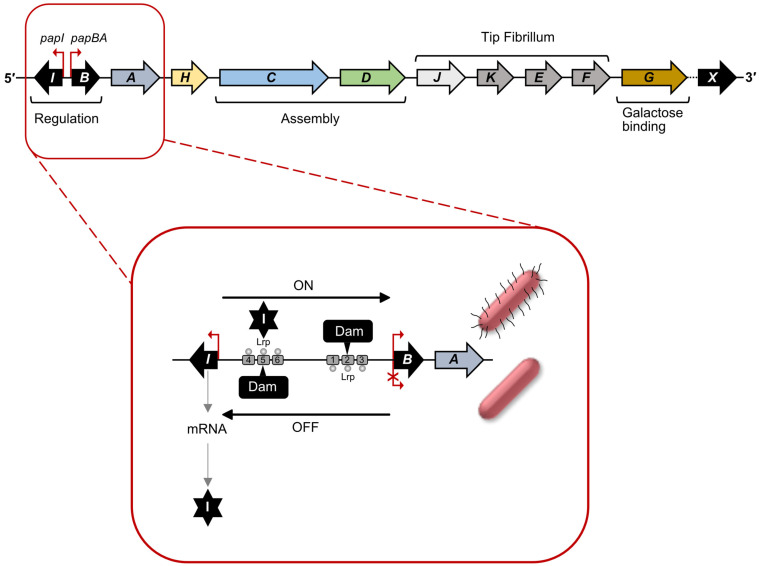
Regulation of Pap pili expression: methylation/epigenetic switch. The *pap* operon, indicating key features. Genes encoding regulatory proteins, PapI and PapB; pilus assembly proteins, PapH, PapC and PapD; tip fibrillum proteins, PapJ, PapK, PapE and PapF; and adhesin protein, PapG are indicated by different shaded boxes and labelled as per function, the regulator PapX is present in some strains downstream from the *pap* operon and downregulates motility. Regulatory network of *pap* operon, as pull-out, depicts the regulatory region of divergently transcribed *papBA* and *papI* promoters. Promoter DNA-methylation states with Dam methylation sites, responsible for ON (piliated) and OFF (non-piliated) are indicated. DNA methylation is blocked by Lrp-binding at sites, 4–6 in Phase-ON cells and at sites, 1–3 in Phase-OFF cells.

Like type 1 fimbriae, genes encoding Pap pili are also clustered (*pap* locus) on the chromosome and controlled by phase variation [18,66] The core *pap* locus contains 11 genes, plus the *mrpJ* homologue, *papX*, located a short distance downstream in some strains [8,43,65]. Transcription of the *pap* locus is controlled by the *papBA* promoter and regulated by a phase variation (ON and OFF) mechanism via the PapB and PapI regulatory proteins [52]. In Pap pili PapA is the major pilin, while PapJ, PapK, PapE and PapF constitute the tip fibrillum. PapG is a galactose-binding adhesin and PapH is the pilus anchor. The other two gene products, PapD (periplasmic chaperone) and PapC (OM, usher), aid in CUP-based assembly of the Pap pili (Figure 4) [65].

Phase variable control of expression of the *pap* locus is different from that of the *fim* locus. In the *pap* locus, the ON-OFF switch is determined by binding of Lrp to two different Dam methylation sites, within the promoter region. Lrp-binding prevents methylation of the site leading to either activation (Phase-ON) or repression (Phase-OFF) of transcription of the *pap* gene cluster. The regulatory proteins, PapI (8 kDa) and PapB (12 kDa), positively control the expression of the *pap* locus in association with the global regulatory proteins, Lrp, CAP and H-NS, as reviewed [18,52]. The intergenic regulatory region (416 bp) between *papI* and *papB* contains six Lrp-binding sites that control transcription of both *papA* and *papB* (Figure 4). Within this, there are two DNA methylase (Dam) sites, GATC^prox^ and GATC^dist^ located within Lrp-binding sites 2 and 5, respectively. When accessible, these sites are methylated at the A base of the GATC motif, but when Lrp is bound, methylation of the sites is blocked. Lrp-binding proximal to the *papBA* promoter inhibits transcription of the *pap* operon, whereas binding at the distal site permits methylation at site 2 and activates transcription of *pap*BA (Phase-ON). Lrp binds with higher affinity to sites 1–3 than to sites 4–6 [52]. The regulator, PapI promotes binding of Lrp to sites 4–6 rather than 1–3, promoting the ON state (Figure 4). Furthermore, *pap* locus transcription is also under the control of catabolite repression, requiring binding of cAMP-CAP complex, 60 bp upstream of the Lrp-binding site. Similar to regulation of type 1 fimbriae, expression of *pap* pili is also controlled by several environmental stimuli [44]. Transcription of *pap* pili is significantly repressed during growth at lower temperatures (<26 °C) in Luria broth. In addition to environmental factors, proteins not belonging to the *pap* locus have been shown to contribute to the positive regulation of the Pap pilus transcription. For example, the two-component sensor-regulator, CpxAR appears to be activated during misfolding of Pap subunits in the periplasm as well as on binding of Pap pilus to solid surfaces and inhibits transcription by binding close to the GATC sites [67,68].

A relatively new study identified the role of a small RNA—*papR*—in the regulation of *pap* locus phase variation, during infection of bladder epithelial cell lines with UPEC, strain UTI89 [69]. Trans-acting *papR* sRNA acts as a post-transcriptional repressor for *papI* and Lrp was shown to act as a transcriptional activator of *papR* expression. Deletion of *papR* increased bacterial adhesion to both kidney and bladder cells in the absence of type 1 fimbriae [69,70]. Involvement of *papR* sRNA in control of production of Pap pili facilitates rapid adjustments in response to changing environmental conditions during infection.

A role of Dam methylation/LRP control in phase variable expression has been demonstrated for a number of other fimbrial clusters [18], including *E. coli afa*/*dra* family of fimbriae clusters and the *E. coli sfa* cluster that encodes S pili (Figure 1) [18,71]. *E. coli* S fimbriae are involved in newborn meningitis and Afa family of fimbriae are common in UPEC strains and form thin surface fibres with an afimbrial appearance [8]. In both cases homologues of PapI and B are involved, AfaF/ AfaA and SfaC/SfaB respectively, and LRP-binding to the PapI homologue promotes expression. Interestingly, analysis of the *afa* locus in certain clinical strains has identified IS elements of varying length between AfaF and AfaA, which in the case of IS1 insertion led to a strong hybrid promoter and enhanced expression [71].

## 3. Other DNA Binding Regulators, Dedicated and Global

### 3.1. pH 6 Antigen, Regulation by a Sensor-Regulator

The FGL-grouped fimbriae are often classified as polyadhesins and show structural and functional differences compared to the FGS-grouped fimbriae [24,47]. The FGL fimbriae are linear polymers of one or two protein subunits. In some cases, the polymer possesses one or two adhesive sites, which are independent of each other and specific to host cell receptors. Thus, permitting polyvalent fastening of bacterial adhesin and host cell receptors that may increase the affinity of binding and enhance adhesion. The chromosomally located *psa* or *myf* locus, which encodes the pH 6 antigen in *Yersinia* spp. is an example of this [50,72,73,74,75]. The pH 6 antigen is expressed at low pH and inside macrophage, hence its name. The *psa* locus (Figure 1) is composed of five genes, *psaE, psaF, psaA, psaB* and *psaC* and encodes a single fimbrial subunit Psa which is assembled to form polyadhesive fibres with two low affinity binding sites for galactosyl residues and phosphatidylcholine [24,50]. Regulation of the *psa* operon appears to be an unusual example among fimbrial systems. The regulators, PsaE (24 kDa) and PsaF (18 kDa), which are required for transcription of *psa*, are both located within the inner membrane. PsaE also possesses a cytosolic N-terminal DNA-binding domain with homology to PhoB and OmpR of *E. coli*, ToxR of *Vibrio cholerae* and HilA of *Salmonella typhimurium* [72]. PsaF appears to be important in stabilising PsaE. The C-terminal domain of PsaE lies in the periplasm and interacts with the periplasmic domain of PsaF [72]. Thus PsaE/PsaF regulation has some structural characteristics of a one component sensor-DNA binding regulator system.

In *Y. pestis*, maximum expression of Psa occurs at 37 °C at pH 6. Understanding the mechanism of sensing temperature and pH is limited. There is evidence that the 5’UTR of *psaE* contributes to temperature sensing by forming a polyU RNA thermometer-like structure at lower temperature [51]. In addition, the central regulator, RovA (18 kDa), has been shown to interact with promoter regions of *psaE* and *psaA* [73,75]. A study by Zhang et al. demonstrated that PhoP and RovA recognise the promoter-proximal regions of *psaEF* and *psaABC* [73]. RovA activated *psaEF* and *psaABC*, whereas PhoP repressed both *psaEF* and *psaABC* through direct association between RovA/PhoP and the target promoter regions. It has been suggested that this reciprocal regulation of *psa* genes by PhoP and RovA could contribute to the tight regulation of pH 6 antigen expression during infection [73,74].

### 3.2. Agg and Aaf Fimbriae, Regulation by A/X Family Regulator, AggR

Enteroaggregative *E. coli* (EAEC) is a diarrhoea-causing pathogen in adults and children [19,76,77,78]. The Aaf I/III and Aaf II fimbriae, encoded on the clusters *agg* and *aaf* shown in Figure 1, form thin bundled fimbriae that lead to a characteristic stacked-brick biofilm of these *E. coli* cells bound to host epithelial cells [76,77]. The operon for the Aaf fimbriae is located on the pAA plasmid and comprises genes encoding a chaperone, *aafC*, an outer membrane usher, *aafD,* and a major and minor pilin subunit, *aafA* and *aafB,* respectively. Expression of Agg and Aaf fimbriae of EAEC is controlled by AggR, an A/X family transcriptional activator, located 9 kbp upstream from the respective fimbrial locus [79]. AggR dimerizes and binds to an AT rich consensus sequence overlapping the promoter to regulate transcription of *agg* fimbrial genes [80]. AggR has also been shown to act as a global regulator [81,82]. Forty-four additional AggR targets, including genes encoding dispersin, a surface protein (Aap), dispersin translocator and Aai type-VI secretion system were identified. AggR was found to activate the expression of Agg fimbriae in response to temperature, oxygen tension, and osmolarity as well as the growth media composition [79]. Five variants of Aaf (Aaf/I-Aaf/V) are known and these variants show a high degree of similarity in the CU assembly genes [83]. The differences are mainly in the genes that encode the major fimbrial structural protein, AggA and AafA. Polymers of these subunits bind fibronectin, only the Aaf/V variant Agg5A lacks fibronectin-binding capacity due to changes in pilin structure [83]. Aaf/II from *E. coli* strain O42 has an unusual cluster organisation with split operons for *aafDA* and *afaB-aafCB* locus that otherwise remain functional (Figure 1) [80,84].

### 3.3. CS and CfaI Fimbriae, Regulation by A/X Family Regulator, Rns

CUP fimbriae produced by enterotoxigenic *E. coli* (ETEC) were initially identified serologically as major components of Colonisation Factor Antigens (CFA) and further characterised genetically as a dominant proportion of Coli Surface (CS) antigens [70]. At least 20 different CUP-assembled CS fimbrial clusters have been identified so far among ETEC strains, although most strains express only two or three types [70]. Many of these, CFA/I, CS1, CS2, CS4, CS14, CS17, CS19 and PCF071, belong to the alternate CUP fimbriae, assigned as the α clade (based on usher phylogeny [37]), and form rigid fimbriae. Both CS3 and CS6 (Figure 1) belong to the FGL group and γ3 clade [24,70]. The most commonly observed fimbriae are CS1-CS6 and CFA/I, of which the mechanism of regulation of CS1, CS2 and CFA/I have been studied in some detail [8,18,70].

The gene cluster of CS1 fimbriae, *cooBACD,* is located on a large plasmid, pCOO and regulated *in trans* by an A/X family transcriptional regulator, Rns [84,85,86]. Rns is encoded on a distinct plasmid, unlinked to any CU system, and is flanked by a transposase encoding gene on one side and pseudogenes on the other [87]. Expression of CFA/I fimbriae is activated by the Rns homolog CfaD/CfaR, which shares 95% amino acid identity with Rns, is functionally interchangeable with Rns and recognises the same binding sites as Rns [18,88]. ETEC fimbriae for which expression is known to be activated by Rns are phylogenetically related and belong to the α clade, CS1, CS2, CS4, CS14, CS17, and CS19 [18,70,89,90,91,92]. There is some evidence that CS3 is under Rns control in a recombinant strain of *Vibrio cholerae* but not in ETEC [18,93]. In addition to activating the expression of fimbrial genes, Rns positively regulates its own gene expression by direct binding at three sites, centred at −227, +43 and +82 (relative to transcription start site) [86,94]. The involvement of downstream binding sites (at +43 and +82) is often associated with transcription repression. However, it has been shown that activation of Rns requires at least one of the two downstream binding sites and the upstream binding site [86]. Rns activates the expression of several putative virulence genes [95,96]. A DNaseI footprinting located the same Rns binding site immediately upstream of the −35 element of P*coo* promoter (required for the expression of CfaA, CS1 fimbriae) and the promoters of CS17, CS19 and PCFO71 fimbriae [85,92], suggesting Rns may activate transcription by direct contact with RNA polymerase. In addition to the binding site near to −35 promoter element, a second binding site was identified further upstream in all cases (at −88 in CfaA, at −144 in CS1, at −109.5 in CS17 and PCFO71 and at −108.5 in CS19). Each site showed an additive effect on Rns-dependent transcription activation, indicating requirement of the distal site also for full activation of Rns [85,86,92].

Rns homologs, linked to regulation of virulence factors, have been identified in several strains of ETEC and other enteropathogenic bacteria. Proteins having strong homology to Rns are CsvR and FapR from ETEC [97], PerA of enteropathogenic *E. coli* [98], AggR of EAEC [79,80], ToxT of *Vibrio cholerae* [99] and VirF of *Shigella* spp. [100,101]. Among these, AggR, CsvR, PerA, and ToxT regulate the expression of fimbrial genes. The VirF of *Shigella* spp. activates the expression of *icsA* and *virB* genes which have roles in invasion and cell-to-cell spread of bacteria in the host epithelial cells, respectively [100].

Transcription activation by Rns and its homologs is thermo-sensitive and is repressed with involvement of H-NS at low temperatures [8,101]. Like most A/X family regulators, Rns is a two-domain protein, with an N-terminal domain (NTD) and a conserved C-terminal DNA binding domain (DBD) with two predicted HTH motifs [102]. A mutagenesis study using pentapeptide insertions demonstrated that Rns uses both HTH motifs to make DNA contacts and thereby activates the expression of CS1 fimbrial genes [102]. In support of this, a uracil interference study by Munson et al. showed that Rns contacts two major grooves of the DNA to activate transcription [85,86]. Like DBD of several A/X family regulators, Rns DBD is believed to be involved in making RNAP contacts, although the region involved in making such contacts has not been identified [85,90].

The role of Rns-NTD was largely unknown. There was no evidence, in vivo, that Rns responds to any effector molecules [91]. However, N-terminal deletion mutagenesis showed that Rns-NTD is essential for transcription activation both in vivo and in vitro [91]. A truncated version of Rns (first 61 amino acids deleted) also resulted in the complete loss of DNA-binding and transcription activation or repression at the corresponding promoter regions. The Rns-NTD motif from I12-M18 is highly conserved among the closest homologs, sharing about 74% amino acid identity compared to 26% identity in the overall NTDs, suggesting a crucial role of this motif in the overall function of Rns. In support of this, Munson and co-workers isolated two random mutants (I14T and N16D) of Rns-NTD and found their activities were decreased dramatically at the *rns* promoter, indicating the importance of I14 and N16 residues in transcription activation [91]. It was not clear whether Rns acts primarily as a monomer or dimer with a role of the NTD domain in dimerisation [91,103] or whether it may be involved in effector ligand binding. Interestingly, recently Rns has been crystallised as a dimer in the presence of decanoic acid, providing evidence that Rns-NTD may sense and respond to a fatty acid ligand [45].

### 3.4. F1 Capsule, Regulation by A/X Family Regulator, Caf1R

**Figure 5:**
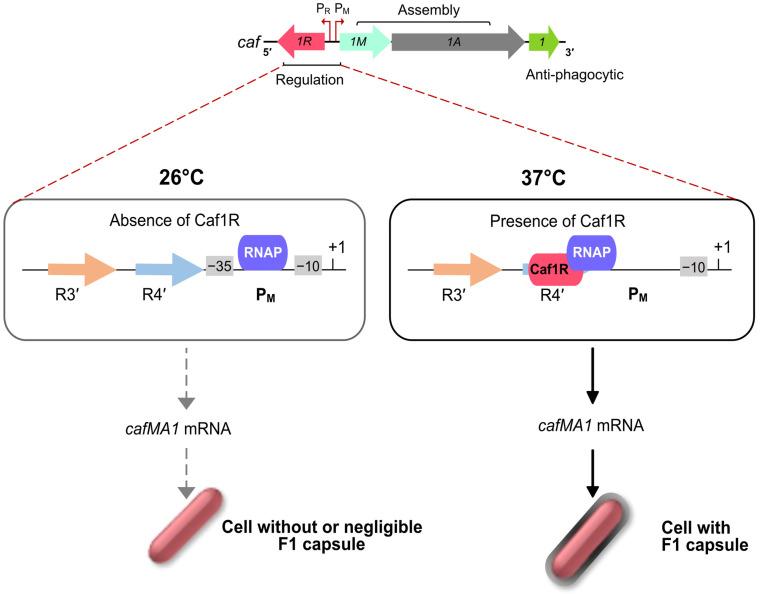
Regulation of expression of *Y. pestis* F1 capsule by A/X family regulator, Caf1R. Caf1R-dependent and independent transcription-regulation of the *caf* operon at P_M_ promoter. It was suggested that interaction of Caf1R with RNA Polymerase at the R4′ *caf* DNA motif at 37 °C is vital to initiate transcription of the *cafMA1* operon at the P_M_ promoter. Expression of the *cafMA1* leads to an abundant level of F1 on the cell surface, giving the appearance of encapsulated cells. At 26 °C, in the absence of Caf1R, there is little or no expression of *cafMA1* hence giving the appearance of non-capsulated cells.

The F1 capsular antigen from *Y. pestis* comprises long, linear fibres of a single subunit (Caf1) and represents an example of a non-pilus organelle assembled via the FGL (γ3) ushers [24,28,37]. The thin fibres of Caf1 subunit collapse around the bacterial cell forming a capsule-like structure that contributes to the anti-phagocytic arsenal of the bacterium [48,104]. Unlike the *psa* locus, the *caf* locus is specific to *Y. pestis*. Initial sequencing of the *caf* locus identified the divergently expressed *caf1R* gene encoding an A/X family regulator, Caf1R [105], but the mechanism of regulation of this key antigen was only more recently elucidated, in 2016, by Kumar, D. [38,49]. Kumar confirmed Caf1R as an essential activator for high level expression of the *caf* operon and capsule formation. Using promoter-*lacZ* fusions and Electrophoretic Mobility Shift Assays (EMSA), the Caf1R binding site was identified overlapping the −35 element of the P_M_ promoter, a characteristic of Class II transcriptional activators (Figure 5) [38,49]. Structure-modelling of a Caf1R-DNA binding site complex confirmed location of a virtually non-functional spontaneous point mutation in Caf1R to DNA binding helix6 [38].

Autoregulation is a common feature among A/X family regulators. Examples of autoactivation include Rns [86,91], PerA [106], MarA [107] and AggR [82]. Unlike *caf1R,* these regulators are primarily ‘unlinked’ to the target operons. Promoter fusion studies also confirmed autoregulation of *caf1R* [38]. F1 fibres are expressed at 37ºC in the mammalian host but not at 26 °C in the insect host (Figure 5) [48]. Temperature regulation of expression has been reported for a number of fimbriae, with involvement of H-NS contributing to repression at low temperatures [58,64]. The untranslated intergenic region of the *caf* locus is known to be required for temperature regulation of Caf1R dependent activation of *cafMA1* [38] but the mechanism by which this occurs remains to be elucidated.

Transcription activation by A/X family regulators, as seen by Caf1R at P_M_ promoter [38,49], is quite common. In many well studied cases, binding of a small metabolite is required for activation [99,108,109,110,111]. Caf1R also possesses a C-terminal ‘sensing’-like domain, but it remains to be investigated if this binds anything or is involved in dimerisation. Bioinformatic analysis of putative and characterised DBDs of 62 A/X regulators grouped Caf1R DBD with regulators controlling metabolic processes [112]. Unexpectedly, it was aligned very distantly to AggR and VirF, regulators of virulence factors including fimbriae. The Caf1R was most closely aligned with the XylR regulator of *E. coli* K-12, which is essential for the transcription of genes required to utilise D-xylose and L-arabinose [111]. Whether this relationship to XylR and other regulators of metabolic processes is an indication of some metabolic process also controlled by Caf1R or is an indication of small molecule sensing by Caf1R remains to be seen.

Caf1R-dependent regulation of divergent expression of the *caf* locus represents a good model for studying the regulation of CUP-fimbriae controlled by a ‘linked’ A/X family regulator. No doubt, there will be many other factors controlling the expression of the *caf* locus. To fully understand the regulation of F1 expression, it will be important to map the role of other cellular factors on the proposed models [38,49]. Virulence of *Y. pestis* is still not well understood, and regulators involved in global regulation may be critically important. Hence, even though Caf1R appears to be a dedicated regulator, it is still important to consider a possible role of Caf1R in trans regulation of other virulence or metabolic factors. Furthermore, there is a lot of interest in the development of attenuated vaccines and heterologous expression of F1 [113,114,115]. In this type of vaccine, it is essential to retain optimum expression of F1. For this reason, a complete understanding of factors controlling the expression of F1 is crucial.

## 4. Conclusions and Perspectives

CUP fimbriae are prevalent in Gram-negative bacteria and are often important virulence determinants, required for adhesion, establishment of colonisation and in some cases invasion. Their structure, regulation and involvement in urinary tract and gastrointestinal infections, in particular, have been studied in detail. CUP fimbriae may be complex structures with a terminal adhesin, or more simple fibres with only one or two subunits which in some cases appear to function as polyadhesins to enhance binding and in others collapse around the cell to form an anti-phagocytic capsule-like structure. Enterobacterial genomes typically include multiple CUP clusters, many of which have different roles during infection. Hence, controlled expression of fimbriae is important to ensure expression in the correct niche while repression, when not required, facilitates evasion of the immune system.

Regulation of expression of fimbrial loci is multi-layered. Fundamentally, phase variation permits a rapid switch from fimbriated cells to non-fimbriated cells, potentially ensuring a guaranteed source of either type of cell. Extensive studies on the recombinase dependent promoter switching of type 1 fimbriae and Dam-methylation of Pap pili have highlighted the significance of global regulator involvement to appropriately moderate phase variation in response to environmental cues, such as temperature, stress, nutritional status [8,18,52,54]. Cross-talk during expression of Pap pili has been shown to contribute to downregulation of type 1 fimbriae. Additionally, an inverse relationship between fimbriae expression and motility is controlled by an additional regulator (e.g., PapX and MrpJ) encoded close to, or part of, the fimbrial cluster [42]. This knowledge provides a starting template to understand control of fimbrial expression in other systems or bacteria where similar regulators of phase variation are identified. But other mechanisms of phase variation of fimbriae expression also exist. Expression of Hif fimbriae of *Haemophilus* is controlled by slipped strand mispairing and variation of the number of residues between the −10 and −35 elements of the promoter, thus leading to phase variable expression of fimbriae [116].

The γ3 clade (FGL group) of fibrillar structures represent a small group assembled by phylogenetically related chaperones and usher proteins [24,37], but a remarkably diverse range of mechanisms of regulation. Expression of the Aaf fimbriae of aggregative *E. coli*, is regulated by an unlinked A/X regulator, AggR, and the *aaf*/*agg* fimbrial clusters are only one example within the AggR virulence regulon. Further studies will clarify how commonly fimbrial clusters are controlled by unlinked A/X regulators and involvement of fimbrial A/X regulators in global regulation. The Afa/Daa/Dra family of fimbriae are closely related varying primarily in subunit sequence [82]. Regulation of *afa* and *daa* loci are known to be dependent on Dam-methylation in a similar manner to the *pap* operon [71]. The pH 6 antigen and F1 capsule of *Y. pestis* also have regulator(s) within the fimbrial cluster, but that of the *psa* locus is reminiscent of a one component-like sensor regulator system [117], while the *caf* locus is controlled by an A/X family regulator, Caf1R [38,49]. These fimbrial loci, acquired at different times during evolution of *Y. pestis* [48] have co-opted different regulatory mechanisms. All of these fimbrial loci are important in infection and disease. A detailed understanding of the primary mechanism of regulation, how this is influenced by relevant environmental factors and, importantly, the interplay between expression of these loci and other virulence determinants during infection and disease will provide a solid basis for development of new intervention strategies. In addition to targeting transcription-regulators, adhesins are well documented immunogens with potential to act as vaccine candidates. In depth understanding of regulatory mechanisms will aid in optimising expression which in turn is important in development of attenuated or whole cell killed vaccines.

## Data Availability

Not applicable.
